# Neuroendocrine Tumors of the Lung

**DOI:** 10.3390/cancers4030777

**Published:** 2012-07-31

**Authors:** Annette Fisseler-Eckhoff, Melanie Demes

**Affiliations:** Department of Pathology und Cytology, Dr. Horst-Schmidt-Kliniken (HSK), Wiesbaden 65199, Germany; E-Mail: Demes@pathologie-wiesbaden.de

**Keywords:** carcinoids, LCNEC, SCLC

## Abstract

Neuroendocrine tumors may develop throughout the human body with the majority being found in the gastrointestinal tract and bronchopulmonary system. Neuroendocrine tumors are classified according to the grade of biological aggressiveness (G1–G3) and the extent of differentiation (well-differentiated/poorly-differentiated). The well-differentiated neoplasms comprise typical (G1) and atypical (G2) carcinoids. Large cell neuroendocrine carcinomas as well as small cell carcinomas (G3) are poorly-differentiated. The identification and differentiation of atypical from typical carcinoids or large cell neuroendocrine carcinomas and small cell carcinomas is essential for treatment options and prognosis. Pulmonary neuroendocrine tumors are characterized according to the proportion of necrosis, the mitotic activity, palisading, rosette-like structure, trabecular pattern and organoid nesting. The given information about the histopathological assessment, classification, prognosis, genetic aberration as well as treatment options of pulmonary neuroendocrine tumors are based on own experiences and reviewing the current literature available. Most disagreements among the classification of neuroendocrine tumor entities exist in the identification of typical versus atypical carcinoids, atypical versus large cell neuroendocrine carcinomas and large cell neuroendocrine carcinomas versus small cell carcinomas. Additionally, the classification is restricted in terms of limited specificity of immunohistochemical markers and possible artifacts in small biopsies which can be compressed in cytological specimens. Until now, pulmonary neuroendocrine tumors have been increasing in incidence. As compared to NSCLCs, only little research has been done with respect to new molecular targets as well as improving the classification and differential diagnosis of neuroendocrine tumors of the lung.

## 1. Introduction

The origin and tumor development of neuroendocrine neoplasms, also called epithelial neoplasms with neuroendocrine differentiation, are discussed controversially, but most researchers published that these tumors arise from Kulchitzky cells (or enterochromaffin cells, which are normally present in the bronchial mucosa) as a part of the diffuse neuroendocrine system comprising single cells or clusters of 4 to 10 cells [1,2,3]. All invasive lung malignancies are composed of approximately 20–25% neuroendocrine tumors (NETs) and 75% non-small cell lung cancer (NSCLC) [1,2,3]. Neuroendocrine tumors were first described as carcinoid tumors by Siegfried Oberndorfer in 1904 and are developed from hormone producing (endocrine) cells which can be found throughout the following body regions:

*Foregut*: Thymus, lung, bronchi, trachea,
*Midgut*: Small intestine, gallbladder, pancreas,
*Hindgut*: Colon, excluding appendix, rectum,

most common in the small intestine (30.4%) followed by the lung (29.8%) [3,4].

The tumors also have properties comparable to those of neurons. Therefore, they are designated as neuroendocrine tumors. But only one third of these tumors are functionally inactive (no hormone production). Neuroendocrine lung tumors are also characterized by secretory abilities to take up and decarboxylate the amine precursors (APUD system cells) [5,6,7]. According to the World Health Organization (WHO) classification 2004, NETs of the lungs share common morphological, immunohistochemical and molecular characteristics and can be divided into three main entities [7]:

Carcinoid tumors (typical (TC)/atypical (AC)),
Large cell neuroendocrine carcinomas (LCNEC),
Small cell carcinomas (SCLC).

These neuroendocrine entities are further summarized into two groups according to their biological aggressiveness:

Well-differentiated low grade (G1) typical and intermediate grade (G2) atypical carcinoids,
Poorly-differentiated high grade (G3) LCNEC and SCLC.

In contrast to typical and atypical carcinoids, LCNEC and SCLC are not closely related to each other regarding genetic and epigenetic characteristics. Contrary to carcinoids, no precursor lesions are known for SCLCs and LCNECs [8,9]**.**

## 2. Preneoplastic/Precursor Lesions

**Figure 1:**
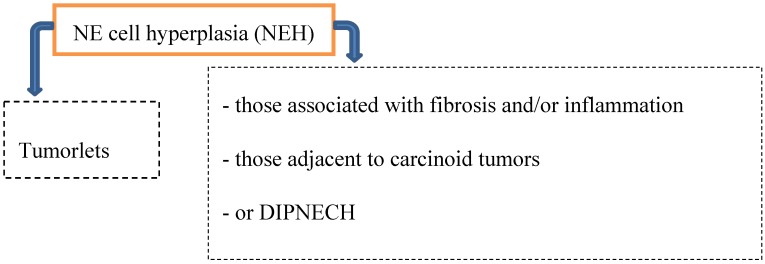
Precursor lesions.

Preinvasive lesions may occur at any age and comprise several types of neuroendocrine (NE) cell hyperplasia (Figure 1):

*Hyperplasia of neuroendocrine cells* of the lung is mainly found in patients with chronic interstitial lung diseases like bronchiectasis, fibrosis and small airway diseases. By light microscopically, neuroendocrine cell hyperplasia includes cells with pale cytoplasma associated with retraction of underlying stroma, or respiratory epithelial intraluminal fingerlike projections [2,6,8].

### 2.1. Tumorlet

**Figure 2:**
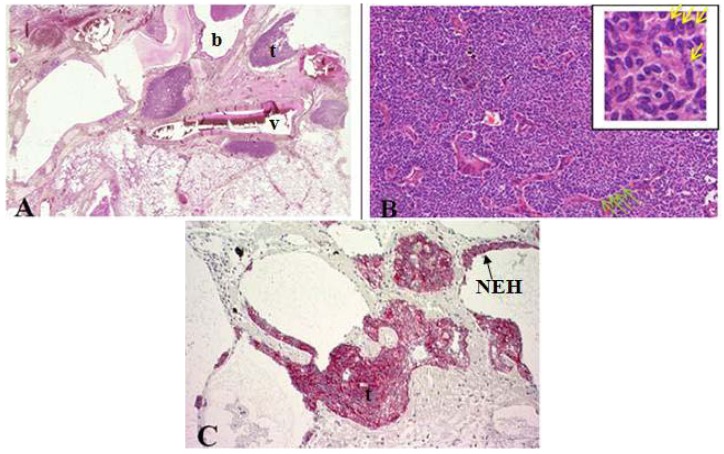
(**A**) Cross section of lung specimen with peribronchiolar fibrosis inducing bronchial enlargement. Tumorlet (t) adjacent to bronchus (b) and vascular (v) tissue; (**B**) High power magnification of tumorlets with neuroendocrine growth pattern, cells are uniform, round with stippled chromatin (yellow arrow); (**C**) NEH with epithelial proliferation and tumorlets immunopositive for synaptophysin.

In general, tumorlets (predominantly in females) are often discovered incidentally at histopathological examination of lung parenchyma and are microscopically defined as peribronchiolar nodular aggregates of uniform, round, oval or spindle-shaped cells with a moderate amount of cytoplasm (Figure 2) [6,10,11]. Peripheral palisading and stippled chromatin of tumor cells can be seen [10]. These lesions are found multiple in lungs of patients with inflammatory processes, fibrosis, tuberculosis, bronchiectasis, around scars and local proliferation in up to 75% of carcinoids which may lead to obliteration of the adjacent bronchiole [1,3,10,11,12].

Tumorlets are often found with surrounding hyalinized, fibrotic stroma [11]. Morphologically, tumorlets are identical to typical carcinoids but smaller in size (≤0.5 cm). Tumorlets should be demarcated from minute meningothelioid nodules (no clinical significance, similar cytologic characteristics), which show no positivity for NE markers and cytokeratins [2,8].

### 2.2. DIPNECH

**Figure 3:**
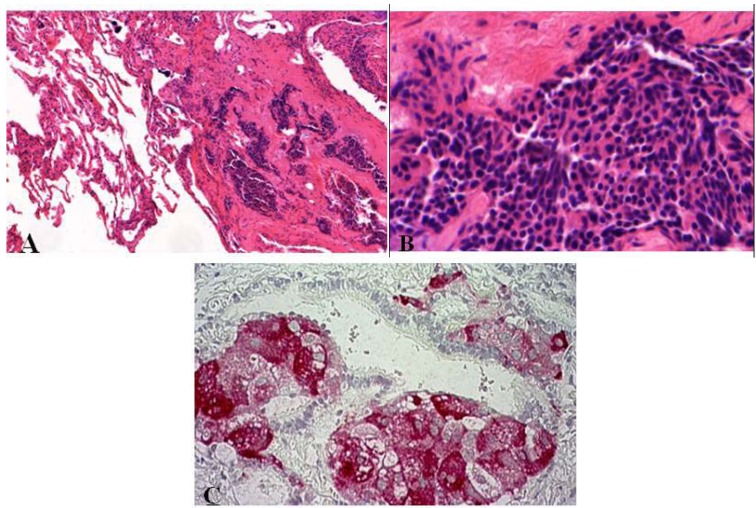
(**A**,**B**) Diffuse idiopathic neuroendocrine cell hyperplasia presented as small nodule aggregates within fibrotic tissue. Small cells with elongated or round nuclei without mitoses or nuclear atypia; (**C**) Synaptophysin stained small nodules of neuroendocrine cell clusters in the epithelium of bronchioli.

As compared to tumorlets, diffuse idiopathic neuroendocrine cell hyperplasias are also associated with airflow obstructions but are rare. Such hyperplasia is characterized by diffuse proliferation of multiple or single neuroendocrine cells presented as small nodules (neuroendocrine bodies) or linear proliferation in the epithelium of bronchioles (Figure 3) [2,8,13,14].

This precursor lesion is often diagnosed when tumorlets are multiple and frequent in the lungs and may coexist [8]. Multiple nodules in unresected lung as often shown by CT (computed tomography) scans can be misdiagnosed as metastases of unknown primary. The differential diagnosis includes tumorlets. DIPNECHS are considered as preneoplastic lesions (WHO 2004) due to the possible progression to carcinoids [1,2,3,8,15].

Multiple tumorlets and DIPNECHs may be seen in the following three settings [1,2,8,15,16]:

*Chronic lung injury* in case of bronchiectasis or fibrosis → progression to carcinoid tumors is typically not seen.
*DIPNECH and the progression to carcinoid tumors* (both AT and TC) has been documented predominantly in women → small airway obliteration or respiratory failure (rare).
*Coexistence of hyperplasia and tumorlets* (often seen in patients with resected carcinoid tumors; 46–76%).

According to Gorthsein *et al.*, metastatic disease (36%) was diagnosed in patients with DIPNECHs [17]. As preinvasive lesions, DIPNECHS as well as tumorlets may progress to carcinoids but not to high-grade neuroendocrine tumors (SCLC or LCNEC).

## 3. Epidemiology

The incidence of neuroendocrine tumors increases linearly [16,17]. Neuroendocrine tumors comprise only 0.5–2% of all malignancies occurring in adulthood [4]. Contrary to patients with SCLC and LCNEC (most are males), patients with carcinoid tumors are significantly younger. Clinically, approximately 20%–40% of patients with typical or atypical carcinoids are nonsmokers, whereas nearly all patients with SCLC (95% of all SCLC arise in the bronchial system) and LCNEC are heavy cigarette smokers [2]. Lung tumors include 1–2.0% carcinoids (AC ~ 0.1–0.2%), 3.0% LCNEC, 15–20.0% SCLC and 75–80% non-neuroendocrine carcinomas [1,2,3,8,18]. Patients with inherited autosomal-dominant syndrome of multiple endocrine neoplasia type I (MEN I) and other hereditary histories show a higher incidence of malignant neuroendocrine lesions [19].

## 4. Clinical Features

The location of the respective tumor and the biological aggressiveness determine the clinical features. In case of centrally located carcinoids recurrent infections, chest pain, cough, dyspnea and pneumonia may occur. Peripherally located ones are generally incidental findings.

Contrary to high-grade NETs, carcinoids arise in around 5% of patients with inherited multiple neuroendocrine neoplasia [2]. Well-differentiated neuroendocrine lesions are capable of producing hormones identical to those of the nervous system, but compared to gastroenteropancreatic NETs, lung carcinoids are only rarely associated with hypersecretion history and paraneoplastic syndromes [2]. The carcinoid syndrome and Cushing’s Syndrome are predominately found in carcinoids and are only rare for patients with LCNECs or SCLCs.

## 5. Clinical Diagnostics

Several imaging procedures can be applied to detect neuroendocrine tumors of the lung:

CT Scan;
Chest X-ray;
Bronchoscopy, Endosonography and Biopsy;
Octreotide Scintigraphy;
Somatostatin Receptor PET.

Radiologically, carcinoids (TC ≤ 2 cm and AC ≥ 4 cm) are presented as nodules or mass (TC with a smooth margin and AC with an irregular margin) which can be misdiagnosed as metastases of a known or unknown extrapulmonary primary tumor. Endobronchial location of these lesions can be well demonstrated by CT. Approximately 30–55% of carcinoids comprise lobar atelectasis, obstructive pneumonitis and partial obstruction [18,19]. Because of their vascular stroma marked enhancement following intravenous administration of contrast can be seen. In about 30% calcification is demonstrated in CT.

In SCLC usually a central mass formed by the combination of primary tumor and lymph node metastases can be found radiologically. Mainly mediastinal lymph node involvement and enlargement is present in most cases (5% to 10% presented as a peripheral nodule without lymph node involvement). Narrowing and displacement of major vessels and bronchi and pleural effusion are common findings [4].

PET (positron emission tomography)—CT plays an important role in the assessment of tumor localization, tumor size and invasion as well as metastasis. Investigation with fluorodeoxyglucose (FDG) may be used for identifying and staging pulmonary neuroendocrine tumors [3]. Low FDG uptake in carcinoid tumors and high-grade NSCLCs can be demonstrated. PET has a low sensitivity in identifying TC tumors. Therefore, other tracers in PET imaging have been evaluated among different studies [3,19]. Another method for identifying metastatic well-differentiated neuroendocrine tumors is somatostatin receptor scintigraphy, which illustrates somatostatin receptors (SSTR) found on tumor cells. The receptors are expressed in about 80–90% of NETs [20]. In contrast to well-differentiated neuroendocrine tumors, undifferentiated ones express SSTR (mainly SSTR_2_ subtype) less frequently (and in lower density) [21]. SST may bind to five different subtypes of specific SST receptors located on the cell surface (SSTR_1_, SSTR_2_, SSTR_3_, SSTR_4_ and SSTR_5_) and acts as an important regulator of endocrine function by inhibiting the secretion of various hormones [20]. Synthetic analogues bind mainly to SSTR_2_, and much less to SSTR_5_. Those synthetic analogues of the peptide hormone somatostatin, octreotide and lanreotide (also in case of inoperable carcinoids), are being the most widely used ones in controlling carcinoid syndrome with metastatic well-differentiated neuroendocrine tumors and are still debated [3,19,22]. Reghi and colleagues compared the immunohistochemical expression pattern of SSTR versus SSTR scintigraphy. They present immunohistochemistry as a less sensitive method as compared to SSTR scintigraphy which can be also explained by tumor heterogeneity [22].

## 6. General Morphological Diagnostic Tools for Identifying and Classifying Pulmonary Neuro-endocrine Lesions

### 6.1. Biopsies and Cytology

Cytology and small biopsy specimens enable an accurate assessment of neuroendocrine tumors. Terada describes a case in which multiple biopsy and cytology of the lung failed to detect carcinoma cells, histological and supportive immunohistochemical examinations helped to diagnose LCNEC of the lung at metastatic site [23]. The differentiation of typical from atypical carcinoids is only done on resected specimens. Because of the high vascularization of endobronchial carcinoid tumor, bleeding under biopsy is often described [2]. SCLC and carcinoid tumor can be diagnosed without problem. Preoperatively LCNEC is most frequently recognized in cytology as NSCLC, not otherwise specified or as ADC [8]. In critical cases, the proliferation index Ki-67 (MiB1) is a useful supportive tool to distinguish low-grade carcinoids from high-grade NE neoplasms.

### 6.2. Histopathological Features of Pulmonary Neuroendocrine Tumors

**Figure 4:**
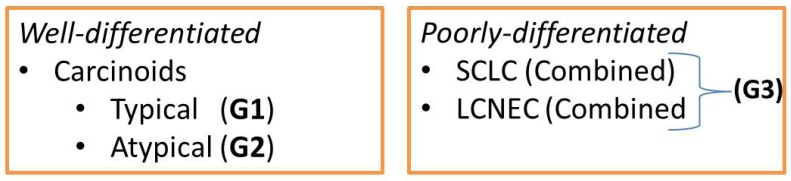
Well- and poorly-differentiated neuroendocrine tumors.

According to the WHO classification 2004, neuroendocrine tumors of the lung can be divided according to the extent of differentiation (well-differentiated/ poorly-differentiated) as follows (Figure 4):

This classification of NETs is based on macroscopic, microscopic and immunohistochemical features. Therefore, the mitotic activity (mitosis/2 mm^2^ = (10 high power fields)) as well as the rosette-like structure, palisading, trabecular pattern, organoid nesting and the proportion of necrosis are common characteristics of pulmonary neuroendocrine tumors [1,3,6,10,13]. Neurosecretory granules can be demonstrated by electronmicroscopy.

Table 1 summarizes additional important macroscopical, histological and cytological features of pulmonary neuroendocrine tumors [1,3,6,8,10,13]. Most common disagreements exist between LCNEC versus SCLC, typical versus atypical carcinoid and atypical carcinoid versus LCNEC [6,8].

**Table 1:** Macroscopical, histological and cytological features of pulmonary neuroendocrine tumors.

NEs	Macroscopy	Histology/Cytology			
		Characterization	Necrosis	Mitosis/10HPF *	Azzopardi effect *
**TC (G1)**	often central, endoluminal grey-yellow endoscopic high vascularization	highly vascularized typical neuroendocrine pattern (*i.e.*, organoid like) relative unimorph, partly granulated cytoplasm moderate nuclear/cytoplasm ratio round, oval, spindle shaped	No	<2	no
**AC (G2)**	often peripheral partly less differentiated	less organoid, more pleomorphic and larger with slightly greater chromatin stained nuclei moderate nuclear/cytoplasm ratio increased cell-atypia round, oval, spindle shaped	possible focal	2–10	no
**Neuroendocrine carcinoma of small cell/intermediate or large cell type (G3)**	less differentiated propagation grey-white often hemorrhage/necrosis	absence of organoid pattern often artifacts of crushing hematoxylin-rich vessel aberrations LCNEC: small nuclear/cytoplasm ratio SCLC: high nuclear/cytoplasm ratio partly free chromatin, big round nuclei, bizarre cell bodies	large areas	>10; LCNEC: often ≥10; SCLC: often >50	LCENC: uncommo n; SCLC: occasional

* HPF = High Power Field; Azzopardi effect describes encrustation of blood vessels with nuclear basophilic material.

### 6.3. Immunohistochemistry

Neuroendocrine markers like chromogranin, synaptophysin and CD56 as well as somatostatin can be a diagnostic tool for discriminating neuroendocrine tumors of the lung. The mitotic count is important to discriminate between low-grade versus high-grade NET and can be supported by Ki-67 proliferation index [24]. Ki-67 may reflect the tumor grade and predicts survival in neuroendocrine tumors, but fail as prognostic marker in some small cell lung cancer patients [25,26].

The mean Ki-67 proliferative index (*p* < 0.001) significantly increases from TC to AC and poorly differentiated neuroendocrine tumors [22]. A proliferation rate of <25% is suggested to designate the NE tumor as SCLC [2,27]. To differentiate LCNEC from basaloid SCLC (p63 and high-molecular-weight cytokeratin positive), 1 NE marker is required [8]. Common immunohistochemical markers are listed in Table 2. Glucose-dependent insulinotropic (GIP) polypeptide receptors are expressed in the majority of bronchial neuroendocrine tumors, and represent a novel target for clinical applications, *in vivo* scintigraphy and targeted radiotherapy [28].

**Table 2:** Common immunohistochemical markers.

Carcinoids	SCLC	LCNEC
Synaptophysin, Chromogranin A, CD56/NCAM, TTF1 (50% are positive + staining weak and focal), Estrogen receptor (50% of carcinoids) *	Synaptophysin, Chromogranin A (only weak), CD56 (most are positive), cytokeratins (AE1/AE3 or CAM 5.2), 34ßE12, TTF-1 (90%)	Synaptophysin, Chromogranin A (coexpressed in 70%), TTF-1 (50%)

* Contrary to central located carcinoids, peripheral ones are more commonly TTF-1 positive. The expression of the estrogen receptor is a pitfall in the differentiation from metastatic breast cancer [2].

### 6.4. Current Staging

Bronchial carcinoid tumors as well as high-grade NE neoplasms are staged by the same criteria as applied to NSCLC (7th edition of the TNM staging system) [20]. For patients with small cell carcinomas who have received neoadjuvant chemotherapy and/or radiation therapy, the quantification of therapy-induced tumor regression provides prognostic relevant information on tumor response and is defined according to Junker and Müller [29,30,31,32]:

– Absence or minimal presence of tumor regression(Regression grade I),
– Incomplete tumor regression (Regression grade II),
– Greater than 10% residual viable tumor (Regression grade IIA),
– Less than 10% residual viable tumor (Regression grade IIB),
– Complete tumor regression, without viable tumor tissue (Regression grade III).

The regression grading (Bochumer Modell) based on resections of treated small cell carcinomas [29,30].

## 7. Carcinoid (Typical/Atypical) Tumors

**Figure 5:**
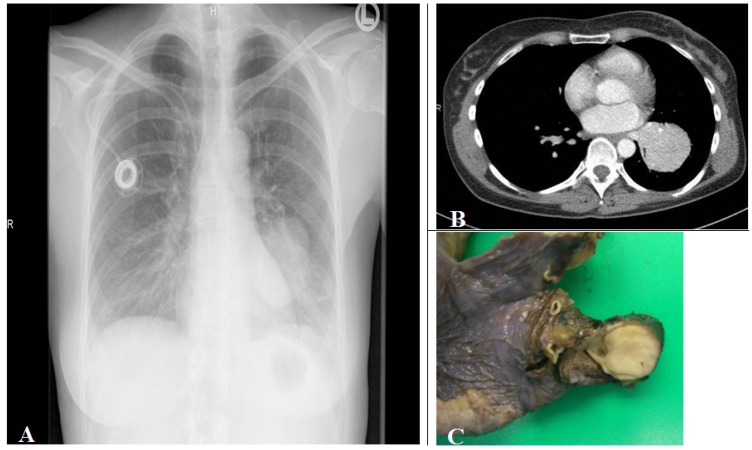
(**A**) Corresponding chest radiograph and (**B**) thorax CT scan (parenchymal window) of a patient with a tissue density/mass (left lower lobe compression) induced by an intrabronchial localized carcinoid tumor demonstrated in the lobe resection tissue (**C**). The tumor is seen as white to yellow tan intrabronchial tissue mass.

Tumors are mainly found (75%) as intraluminal centrally located well demarcated tan to yellow colored tumors within a large central bronchi (Figure 5).

25% of carcinoid tumors are peripherally located as intraparenchymal coin lesion without an association to a bronchus [33]. Endoscopically, the tumor is covered by an intact or ulcerated overlying bronchial mucosa, which often may induce squamous cell metaplasia. Biopsy may induce high vascularization bleeding.

**Figure 6:**
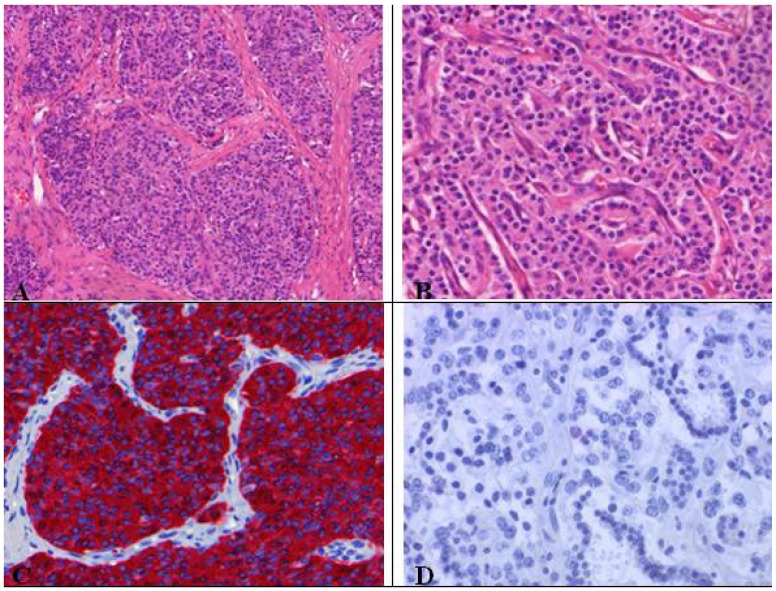
(**A**) Typical carcinoid with organoid growth pattern and (**B**) trabecular pattern with delicate intervening vascular stroma; (**C**) Neuroendocrine differentiation demonstratedby chromogranin positivity; (**D**) Absence of proliferation activity (MIB-1 ≤ 1%), no mitoses.

As visible in Figure 6, typical carcinoids (G1) are characterized by organoid growth patterns suggesting neuroendocrine differentiation indicated by trabecular, insular, palisading, ribbon and rosette-like arrangements. The tumor cells are uniform, polygonal with finely eosinophilic cytoplasm, nuclei with a fine granular chromatin pattern (pepper salt morphology), inconspicuous nucleoli and a scant to moderate amount of cytoplasm [8]. Necrosis is absent. Highly vascularized fibrovascular stroma, hyalinization of stroma, cartilage or bone formation as well as amyloid can be present. The absence of necrosis and only1 mitosis per 10 HPFs designate the NE tumor as TC [8].

**Figure 7:**
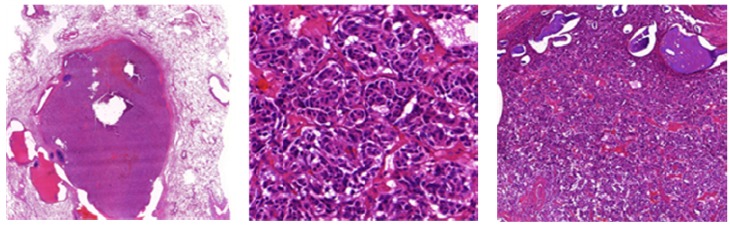
Atypical carcinoid tumor with vascularized stroma, focal necrosis and more than 2 mitosis/2 mm².

**Figure 8:**
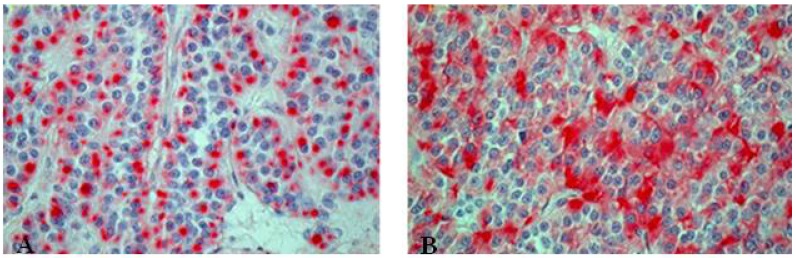
Immunohistochemical staining of atypical carcinoid tumor with (**A**) punctual positive KL1 reaction and (**B**) chromogranin positivity.

Atypical (G2) and typical (G1) carcinoids present very similar morphological and cytological characteristics (Figure 7). In contrast to typical carcinoids, atypical ones represent 2–10 mitoses per 2 mm^2^ or 10 HPFs and often centrally located punctate necrosis similar to comedonecrosis. AC show greater nuclear polymorphism than seen in TC comprising nucleoli and nuclear membrane irregularities, but this is not part of the diagnostic criteria of AC [2,8]. Atypical carcinoids do not express all NE markers (Figure 8) [2]. For instance, 80% of carcinoids are cytokeratin positive [2,8], which limits the possibility to differentiate between spindle cell carcinoids of mesenchymal tumors and nested carcinoids of paraganglioma (S-100 positivity) [2].

The differential diagnosis of carcinoid tumors include [1,2,8]:

Carcinoid tumorlets;
High grade neuroendocrine tumors:
  Large cell neuroendocrine carcinoma,
  Small cell carcinoma;
Adenocarcinoma;
Mucoepidermoidcarcinoma (glandular pattern) adenoidcystic carcinoma;
Paraganglioma;
Glomus tumor.

## 8. LCNEC

LCNECs are located

→ peripherally (~84%) (not accessible to the bronchoscope);
→ centrally (~16%) with endobronchial tumor growth (tan-colored polypoid mass infiltrating the airway lumen).

The biological behavior concerning aggressiveness is similar to small cell lung carcinoma, but in contrast to SCLC the response to chemotherapy is bad, therefore surgical treatment is acquired.

**Figure 9:**
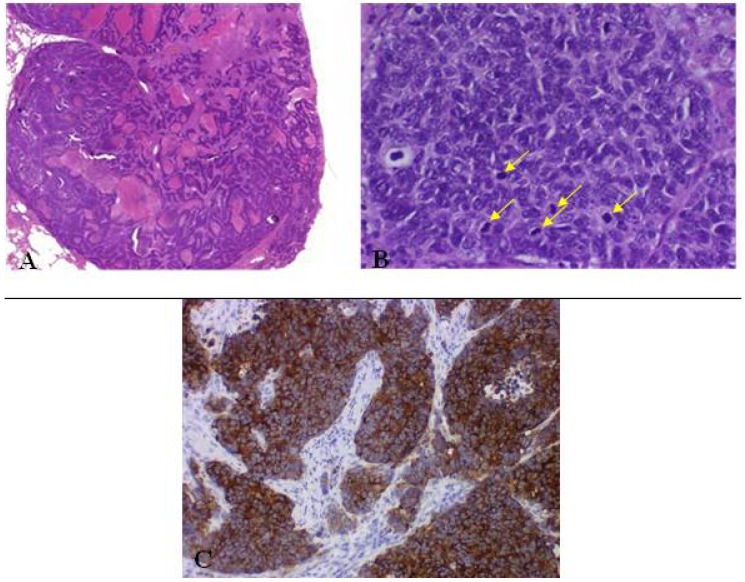
(**A**) Large cell neuroendocrine carcinoma with organoid nesting, trabecular growth, perilobular palisading patterns; (**B**) Tumor cells are large, nucleoli are frequent, high mitotic count (yellow arrow); (**C**) Positive staining of large cell neuroendocrine carcinoma with chromogranin.

LCNECs comprise typical histological features of carcinoid tumors (Figure 9) suggesting neuroendocrine differentiation (WHO):

→ Organoid nesting, trabecular growth, rosettes and perilobular (peripheral) palisading pattern, large zones of necrosis (comparable to SCLC), large tumor cells, usually abundant eosinophilic cytoplasm, prominent and frequent nucleoli (contrast to SCLC, but not always, therefore the cell size should not be used alone to differentiate between SCLC and LCNEC), mitotic counts are typically ≥11 per 2 mm² of viable tumor, at least one neuroendocrine marker should be positive. 50% of LCNEC are positive for TTF1 [8].

Important:

Large cell carcinomas have to be differentiated from SCLC, typical carcinoids and NSCLCs, which has an influence on therapeutic treatment options and prognosis.

### 8.1. Combined Large Cell Neuroendocrine Carcinoma—What Does This Mean?

Large cell neuroendocrine carcinomas with components of:

→ adenocarcinoma,
→ squamous cell carcinoma,
→ giant cell carcinoma and/or spindle cell carcinoma.

A small percentage of LCNEC is heterogeneous and should be treated like other NSCLC.

### 8.2. Differentiation LCNEC *Versus* NSCLC with NE Differentiation (NSCLC-NED)—Always Possible?

NSCLC can have cytologic characteristics which may overlap with LCNEC like prominent nucleoli, vesicular chromatin, extensive necrosis and a high mitotic rate (in undifferentiated NSCLC).

Solid or cribriform nests with focal palisading in some poorly-differentiated adenocarcinomas may mimic LCNEC


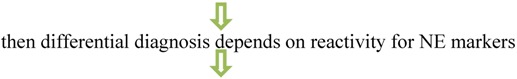


Problem: 10–20% of NSCLCs (more commonly in ADC/SCC) label for NE markers regardless of the growth pattern and designated as


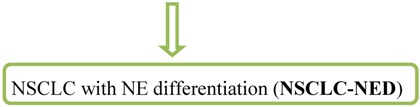


NSCLC with NE differentiation is highly controversially discussed. The other way around, tumors which show LCNEC morphology but no expression of a neuroendocrine marker are designated as


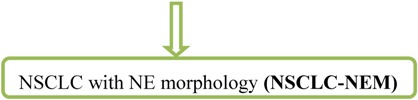


These cases are very rare and their clinicopathologic characteristics are not well established. NSCLC-NEMs are suggested to have a poor prognosis similar to LCNEC [8].

## 9. SCLC

**Figure 10:**
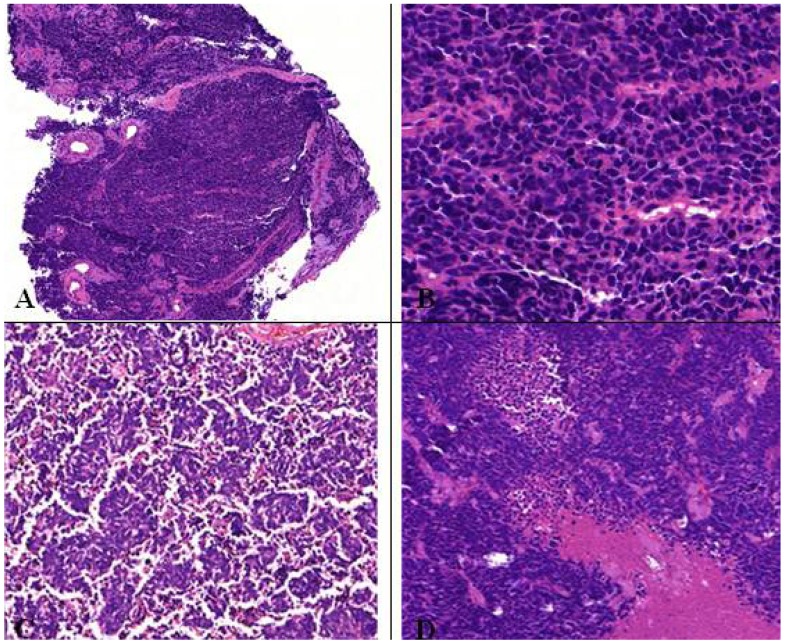
(**A**,**B**) Microscopic feature of small cell lung cancer infiltrate in a bioptical obtained specimen with high mitotic rate. (**C**,**D**) Small cell lung cancer in higher magnification with malignant small cells with scant cytoplasm and defined cell borders, fine granular nuclear chromatin, absent or inconspicuous nucleoli, focal or extensive necrosis.

The majority (~90%) of these high-grade (G3) small cell lung cancers are centrally (lobar or main bronchus) located and often infiltrates the adjacent interstitium. Only bioptical HE stained specimens (with mucosa and tunica propia), occasionally transbronchial biopsy, should be obtained to get representative tumor tissue. When increasing in size, the adjacent parenchyma and peribronchial lymph nodes are involved. The histological diagnosis on small biopsies is in accuracy in about 90% and interobserver agreement among pathologist is about 95% based on the following microscopic features (Figure 10):

→ Neuroendocrine growth pattern (organoid growth, rosettes, peripheral palisading, and occasional pseudopapillary pattern) is seen. In some cases more epitheloid and cuboidal cells or trabecular architecture are found: loose and irregular (or syncytial) clusters and individual, usually small tumor cells (linearly arranged, round, oval, and spindle shaped), comparable with the size of two to three times a mature lymphocyte. Prominent nuclear molding and fragility of the malignant nuclei as well as extensive necrosis is demonstrated. Similar to LCNEC, mitoses (in practice ~50–60 mitoses/2 mm²) can easily be seen. High nuclear/cytoplasmic ratio (scant cytoplasm) as well as typical “salt and pepper” chromatin is shown. Pulmonary neuroendocrine markers (synaptophysin, chromogranin) are only positive in around 70% [8].

As differential diagnosis lymphoid infiltrate has to be excluded and should be taken into consideration supported by immunohistochemistry. In case of extrapulmonary located metastatic SCLCs of unknown primary site, the lung as origin organ should be taken into account. A problem is that the cell size of SCLCs may vary significantly and cannot be used as the main criteria to differentiate those tumors from LCNECs. Thus, in case of larger nuclear size and occasionally more evident cytoplasma the tumor is designated as: 


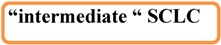


### 9.1. Differentiation of SCLC *Versus* LCNEC—Always Possible?

The most reliable microscopic features for distinguishing SCLC from LCNEC are:

classic nuclear morphology, lacking prominent nucleoli and prominent cell;
membranes.

SCLCs may mimic lymphoma, basaloid squamous cell carcinoma, small cell variant of squamous cell carcinoma, LCNEC, poorly-differentiated NSCLC, merkel cell carcinoma and primitive neuroectodermal tumors (PNET) which can be excluded by supportive immunohistochemistry.

According to the WHO 2004, SCLC can be classified as: *combined small cell lung cancer*.

About 20%–30% of SCLCs are combined small cell carcinomas, 70%–80% are pure SCLC [34,35].

### 9.2. Combined Small Cell Lung Cancer—What Does This Mean?

If the tumor is comprised of only a minor NSCLC component including those of

squamous cell carcinomas, adenocarcinomas, large cell carcinomas,
spindle cell or giant cell carcinoma (less common),

the classification “combined small cell lung cancer” should be applied but the patient has to be treated like a patient with pure small cell lung cancer. If there is no tumor response, a change of chemotherapy to a NSCLC-treatment regime or, if possible surgery should be discussed. Thus, the NSCLC component can be treated by operation after neoadjuvant chemotherapy. Postmortem, 50% of patients with neuroendocrine tumors which were previously designated as pure SCLC show NSCLCs alone or in combination with SCLC after treatment [2,36,37].

### 9.3. Criteria for Combined Small Cell/Large Cell Carcinoma?

**Figure 11:**
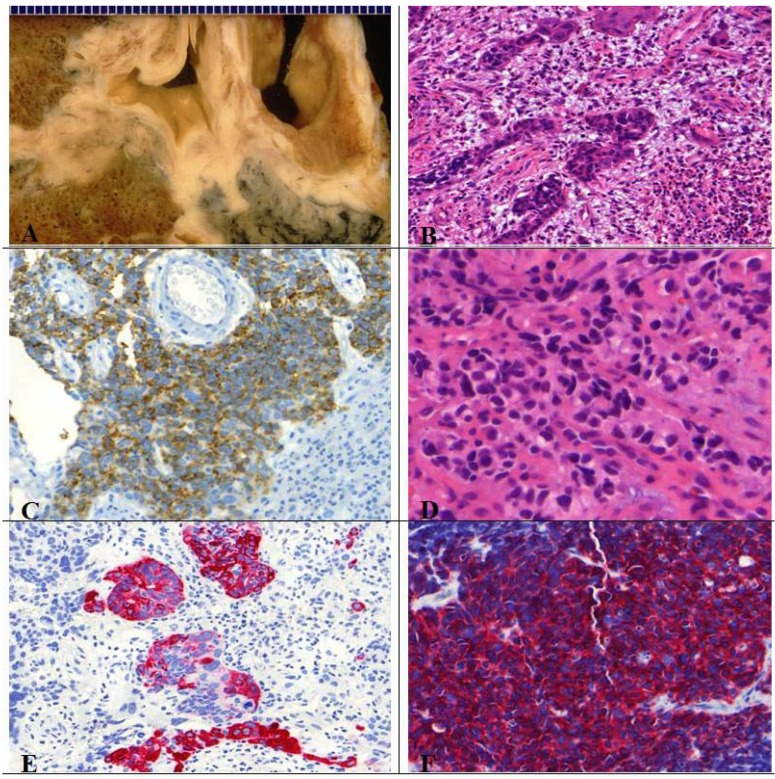
(**A**) Centrally located combined SCLC with histological features of a large cell carcinoma; (**B**) Presence of typical necrosis and (**D**) partly oval, spindle shaped and partly round small cells with mitotic figures. Immunopositive for (**C**) CD56, (**F**) synaptophysin and (**E**) CK7.

At least 10% large cells should be present. Generally defined as a tumor in which large cells, including prominent nucleoli and cell boarders rather than discrete clusters are found (Figure 11) [8]. The prognosis and treatment options of combined SCLC are discussed controversially [2]. Contrary to pure SCLCs, the sensitivity to chemotherapy seems to be decreased for combined SCLCs [2,38].

## 10. Current Treatment and Prognosis of Neuroendocrine Tumors of the Lung

The clinical behavior of these neoplasms varies in dependency of the malignant degree from indolent (TC) to rapidly fatal (SCLC). Differentiating ACs from TCs or LCNEC and SCLC is clinically important due to the variable treatment options and prognoses [5]. The 5-year survival rate ranges from around 87% (average) for patients with carcinoids to 2% for patients with poorly-differentiated tumors (Table 3) [1,2,33,39,40].

**Table 3:** 5-Year survival rate and treatment options ranging from low to high grade NEs [1,2,39]

	5-Year survival rate	Treatment
Typical Carcinoid ( *G1*)	92–100%	surgical resection/lobectomy
Atypical Carcinoid (*G2*)	61–88%	surgical resection/lobectomy
LCNEC (*G3*)	16–57%	cisplatin based chemoth.?/surgery
SCLC (*G3*)	~2–5%	etoposide/cisplatin or carboplatin combination; early stage patients undergo surgical resection

According to Johnson *et al.*, histology and not lymph node involvement predicts long-term survival in patients with pulmonary carcinoids [41]. The preferred treatment options for carcinoids are [3]:

→ lung conserving and radical resection including lymph node dissection;
→ anatomic resection (pneumonectomy or lobectomy)—60–70% of cases;
→ sleeve resection (for centrally located carcinoid tumors);
→ lung sparing procedures (wedge resection, segmentectomy, or sleeve resections).

In typical carcinoids regional lymph node metastases can be found in 10–15% and distant metastases in 3–5% [2,42]. In atypical carcinoids nodal metastases are found in 50% and distant metastases in 25% [2]. As metastases of G1 and G2 NETs are not sensitive to chemotherapy, operation remains the first choice of treatment in metastatic diseases. Due to the high metastatic risk (in 50% to 80%) of these tumors, prophylactic cranial radiation is usually done. Currently, no consensus on clinical management and therefore no treatment guideline for LCNEC exists [8]. Regarding palliative chemotherapy for advanced LCNEC, treatment similar to SCLC is more appropriate than NSCLC [43]. According to Kenmotsu and colleagues, nedaplatin plus irinotecan is effective for LCNEC patients [44], a multimodality management seems necessary [45].

Additional treatments currently under investigation include chemotherapy and radiation, in particular for patients diagnosed with unresectable or metastatic disease at the time of the initial presentation. Responses to standard chemotherapy in prior studies have been poor [3]. Following therapeutic agents like *interferon-α*, *etoposide-based regimes*, *streptozotocin*, *5-fluorouracil and somatostatin analogues *are tested [2].

The best overall response rate to chemotherapy with or without radiation was only 22%, as measured by serial imaging [3]. Currently, further investigation is concentrated on anti-angiogenetic treatment with VEGF inhibitors (bevacizumab) and mTOR inhibitors (RAD001, everolimus). Becacizumab is tested in phase 1 and phase 2 clinical trials alone and in combination with pegylated interferon-α-2b and seems to have efficiency in advanced carcinoid tumors [3]. Everolismus is a novel oral inhibitor of mTOR, a conserved serine/threonine kinase regulating cell cycles [3]. Sunitinib is a multitargeted oral tyrosine kinase inhibitor which is studied in the treatment of advanced carcinoid tumors [3].

Functional CT scan indicated decreases in whole tumor flow, blood volume and permeability surface with anti-angiogenic agents. Radiotherapy as an adjuvant therapy for carcinoid tumors may lead to possible benefits, particularly in patients with lymph node-positive disease. Definitive radiotherapy without chemotherapy has not been shown to improve survival [3]. Palliation with radiation may play a role in treatment of unresectable disease [3]. According to Abedalla and colleagues, results suggest that perioperative chemotherapy may be beneficial in patients with resected SCLC and LCNEC [46].

## 11. Molecular Pathology

In contrast to NSCLCs, neuroendocrine tumors do not often present activated members of the tyrosine kinase family of proteins, such as platelet-derived growth factor (PDGF) or c-Kit and c-Met. Therefore, members of the tyrosine kinase family of proteins seem to play no important therapeutic role till now but have a prognostic value. According to de Pas and colleagues, a patient with LCNEC harboring EGFR mutation benefits from a gefitinib therpay [47]. The overexpression of the Myc family was described in SCLC cell lines and correlates with poor prognosis [8,48,49,50].

Chromosomal aberrations are more likely seen in neuroendocrine carcinomas. Such structural changes of chromosomal sections are more common in high-grade neuroendocrine carcinomas and atypical carcinoids than typical well-differentiated ones. Many of the genetic differences between low-grade and high grade neuroendocrine tumors can be assigned to smoking history, more often seen in high grade tumors [1,51]. P53 mutations, which are correlated with tobacco consumption, are very frequently present in SCLCs and LCNECs [51]. Mutations of MEN 1 gene are restricted to carcinoid tumors [51]. MEN1 is an autosomal dominant genetic disorder on chromosome 11q13 (LOH = loss of heterozygosity) associated with tumors of multiple endocrine organs. Allelic loss of 11p13 locus of MEN 1 occurs in patients with MEN1 Syndrome but also in patients with nonfamilial carcinoid tumors. The MEN 1 gen encodes the protein menin, which should act as a tumor suppressor protein in JunD activation pathway. Deletion of 11q was found by comparative genomic hybridization in 47% of typical carcinoid and in 66% of AC [3,50,51]. The 11q deletion was rarely identified in poorly differentiated lung neuroendocrine cancers (SCLC, LCNEC). [3]. Atypical carcinoid, but not typical carcinoids may display loss of 10q and 13q material [48,49]. Large cell neuroendocrine carcinoma—has specific genetic characters similar to SCLC: allelic losses of 3p21, FHIT, 3p22–24, 5q21 (more frequent in SCLC than in LCNEC), 9p21 and the RB gene. The Retinoblastoma inactivation and loss are typical of high-grade neuroendocrine tumors, whereas typical carcinoid retain RB expression and 20% of atypical carcinoids show loss of RB expression.

Angiogenetic (VEGF, PDGF mTOR) pathways have received renewed attention and enthusiasm. According to Sartelet *et al.*, the vascular endothelial growth factor (VEGF) is highly expressed in 25% of carcinoid tumors [49]. Especially the VEGF/SEMA3F/NP pathway has gained more attention. Kusy et al described the balance between SEMA3F (ligand of VEGFR receptor 2) and VEGF, which may predict the cell motility and apoptotic preservation with respect to the biological aggressiveness of the pulmonary neuroendocrine tumor [52]. In contrast to carcinoids, SEMA3F is lost in high-grade neuroendocrine tumors [2,3,52].

Non-coding RNAs such as miRNAs have gained renewed attention and are controversially discussed as possible prognostic or therapeutic markers [53,54,55]. Additionally, those small molecules are investigated for discriminating between carcinoid tumors and large cell neuroendocrine lung tumors or between large cell neuroendocrine tumors and small cell carcinomas of intermediate cell type, which is often a diagnostic problem for pathologists. High expression of Excision Repair Cross Comlement Group 1 (ERCC1) in typical carcinoids might be predictive for platin-based therapies and ERCC1 seems to have a prognostic impact in lung carcinoids [26].

The prognostic feature of patients with neuroendocrine tumors has to be examined more intensively to find a profile of specific molecular genetic abnormalities in individual tumor correlating with implications for potential therapeutic trials and adjuvant chemotherapy. Common markers involved in tumor proliferation, cellular growth, apoptosis, angiogenesis and metastatic potential might be potential targets for therapy.

## 12. Conclusions

In some cases it is difficult to differentiate between the neuroendocrine tumors of the lung, atypical versus typical carcinoid and carcinoids versus small cell carcinoma and versus large cell carcinoma, but sometimes also the demarcation of NEs from non-NEs is a challenge. There is no currently available immunohistochemical or molecular marker that reliably supports the differentiation of LCNEC from SCLC and NSCLC-NED. Additionally, combined tumors are discussed controversially regarding the treatment options and prognosis. Thus, misdiagnoses may result in wrong chemotherapeutical treatment options. In case of no chemotherapeutical response to current therapy, further biopsies should control the histological classification. Only few studies exist concerning these tumors and further research is needed to clarify the role of potential targeted therapies and to improve the differential diagnosis of these diverse and clinically challenging tumors.
